# Supplementary material for: Ferritin, blood urea nitrogen, and high chest CT score determines ICU admission in COVID-19 positive UAE patients: A single center retrospective study
Source: PLoS One. 2022 Jul 19;17(7):e0269185. doi: 10.1371/journal.pone.0269185 (PMC9295942; doi:10.1371/journal.pone.0269185)
Supplement: S1 Table — (DOCX) [file pone.0269185.s001.docx]

**Table S1.** Pre-existing comorbidities among the COVID-19 positive patients demarcated into non-ICU and ICU admitted**.**

| **Comorbidities** | **non-ICU admitted**  **N (%)** | **ICU admitted**  **N (%)** | **p value** |
| --- | --- | --- | --- |
| No known Comorbidities | 12 (30) | 15 (23) | - |
| Hypertension | 20 (50) | 44 (67) | 0.104 |
| Diabetes | 16 (40) | 35 (53) | 0.231 |
| Cardiovascular | 6 (15) | 21 (32) | 0.066 |
| Chronic Kidney disease | 8 (20) | 18 (27) | 0.488 |
