# Supplementary material for: Ferritin, blood urea nitrogen, and high chest CT score determines ICU admission in COVID-19 positive UAE patients: A single center retrospective study
Source: PLoS One. 2022 Jul 19;17(7):e0269185. doi: 10.1371/journal.pone.0269185 (PMC9295942; doi:10.1371/journal.pone.0269185)
Supplement: S2 Table — (DOCX) [file pone.0269185.s002.docx]

**Table S2.** Treatment or clinical management for COVID-19 positive patients demarcated into non-ICU and ICU admitted.

| **Clinical Management** | **non-ICU admitted**  **N (%)** | **ICU admitted**  **N (%)** | **p value** |
| --- | --- | --- | --- |
| Antiviral therapy | 20 (50) | 62 (94) | **<0.0001** |
| Glucocorticoid therapy | 9 (22.5) | 54 (82) | **<0.0001** |
| Antibiotics | 32 (80) | 63 (95) | **0.019** |
| Continuous replacement therapy | 1 (2.5) | 6 (9) | 0.250 |
