# Supplementary material for: Ferritin, blood urea nitrogen, and high chest CT score determines ICU admission in COVID-19 positive UAE patients: A single center retrospective study
Source: PLoS One. 2022 Jul 19;17(7):e0269185. doi: 10.1371/journal.pone.0269185 (PMC9295942; doi:10.1371/journal.pone.0269185)
Supplement: Attachment — Submitted filename: Response to reviewers.DOCX [file pone.0269185.s003.DOCX]

PONE-D-21-26443

Reviewer #1:

While this is an interesting study the sample size, especially of sub analysis are very small.

**Answer:** We thank the reviewer for the constructive comments regarding the study and agree that the sample size might be small to derive solid conclusions. In order to address the sample size limitation, we included around 50 additional cases in the ICU admitted group from the single center. In total, the current study has 66 in ICU admitted group (compared to the earlier number of 17) and 40 in non-ICU admitted group. (Accordingly, modifications were made in Page 4 lines 94-95 and Page 6 lines 142-149 in the revised manuscript)

As a single center study the generalized applicability of these observations is limited.

**Answer:** We thank the reviewer for the comment. However, in most of the studies and more specifically in relation to COVID-19, clinical characterization from single center study was helpful in determining the factors associated with COVID-19. The data from single-center based studies correlated with other large cohort studies.

For example the observations made in a small cohort of 60 COVID-19 patients in Chinese study (<https://journals.lww.com/md-journal/Fulltext/2021/07300/A_retrospective_analysis_from_a_single_center_for.37.aspx>) were concordant with those obtained from large cohort of 1296 patients from Brazil (<https://journal.einstein.br/article/clinical-characteristics-and-outcomes-of-covid-19-patients-admitted-to-the-intensive-care-unit-during-the-first-year-of-the-pandemic-in-brazil-a-single-center-retrospective-cohort-study/>).

In addition, meta-analysis conducted on 58 independent studies on COVID-19 showed the similar findings for the biochemical parameters from the current study (<https://www.ncbi.nlm.nih.gov/pmc/articles/PMC8444810/>) indicating that the results from multiple centers were in concordance with the present study from the single center. Accordingly, few statements were added in the discussion in Page 16 lines 386 to 388.

In fact, in the present study, the outcome observed in the earlier small cohort (with only 17 ICU admitted cases) were similar to the current larger cohort of 66 cases. Moreover, the accuracy for predicting the ICU admission increased to 94.2% with inclusion of additional 50 cases in ICU admitted group for cumulative radiological and biochemical parameters than the earlier cohort. The conclusions mostly remained same in both the cases (earlier and now) indicating CORADS along with ferritin and blood urea nitrogen values determines the ICU admission more accurately than considered independent (These changes can be seen in Pages 12 and 13 lines 286 to 303).

Some of the sub study groups have only 2 subjects and several recent publications on the same have 500 plus subjects available for analysis.

**Answer:** We thank the reviewer for the above comment. Based on the reviewer comments we believe that they are referring to the Intermediate CORADS group in the ICU admitted cases in Table 1 (Page 6). The score observed in ICU admitted group is mostly greater than 4. From the articles related to CORADS it is understood that score 5 is highly significant in predicting the positive PCR outcome among COVID-19 affected patients (<https://www.ncbi.nlm.nih.gov/pmc/articles/PMC7880207/>, <https://pubs.rsna.org/doi/full/10.1148/radiol.2020201473>). In general, most of the COVID-19 infections show a CORADS score > 4. However, in the current cohort, only 2 out of 66 ICU admitted patients exhibited an intermediate CORADS score. This study emphasizes that along with high chest CT scores, it is important to correlate the biochemical factors in order to predict for ICU admission.

Reviewer #2: This manuscript by Bendardaf and Bhamidimarri et al., proposed predictive metrics for evaluating whether patients will be admitted to the ICU based on biochemical and radiological findings. This is a retrospective analysis. These findings are of importance and global relevance and may be useful for informing medical decisions in some cases.

**Answer:** We thank the reviewer for taking time to thoroughly review the article and identify the importance of the study.

The main concern is the small sample size (N=57). To their credit, the authors acknowledge this limitation in the manuscript, but the broader implication for such a small sample size is the potential to over-train their analysis.

**Answer:** We thank the reviewer for the above comment. We do agree about the sample size issue and hence attempted to address it by collecting information from additional 50 cases from ICU admitted group. In total, the current study has 66 in ICU admitted group (earlier it was 17) and 40 in non-ICU admitted group. The manuscript was revised accordingly as show in in page 4 lines 94-95 and Page 6 lines 142-149.

Essentially, if all 57 patients were used in the creation of the ROC curves, how do we know whether the identified metrics will be any good in the real world? ROC analysis is best when there is a "discovery" step followed by a "validation" step. With such a small sample size, it appears the authors have only done the "discovery" step.

**Answer:** We thank the reviewer for the comment. We agree that the small sample size may not reflect real world data. Thus, we included additional 50 cases in ICU admitted group and performed the analysis on the new set of patients. The biochemical inferences correlated with the earlier analysis applied to the smaller cohort of patients. The important findings based on the larger sample size showed that the cumulative use of CORADS along with ferritin and blood urea nitrogen values to determine ICU admission holds true even when increasing the samples size. In fact, the current cohort showed an increase in accuracy to 94.2% when all the three parameters were combined to determine ICU admission as stated in lines 286-303 in pages 12 and 13 in the revised version. This indicated that the observations made in 57 samples were also relevant in 106 samples and are in coherence with the biochemical interpretations from other such studies performed at different centers (<https://www.frontiersin.org/articles/10.3389/fcimb.2021.632965/full>, <https://bmcinfectdis.biomedcentral.com/articles/10.1186/s12879-021-05840-2>, <https://www.sciencedirect.com/science/article/pii/S1319016421001559>).

Can the authors reach out to another medical center to evaluate whether their patient data can be tested to validate the metrics identified here? This information might also be available from mining in the literature. The risk here is that these metrics might only be relevant to these 57 patients at University Hospital Sharjah.

**Answer:** We thank the reviewer for the comment. In order to assess the findings in other study, we attempted to include data from other medical hospitals for COVID-19, however the information on all the variables were not available to pair the samples with corresponding CORADS score and biochemical characteristics. Thus, we mined the literature to obtain similar data from independent studies and found that most of the observations made in our study reflected in other COVID-19 related studies which were also discussed in the article to correlate the findings from similar studies in another cohort.

For example the observations made in a small cohort of 60 COVID-19 patients in Chinese study (<https://journals.lww.com/md-journal/Fulltext/2021/07300/A_retrospective_analysis_from_a_single_center_for.37.aspx>) were concordant with those obtained from large cohort of 1296 patients from Brazil (<https://journal.einstein.br/article/clinical-characteristics-and-outcomes-of-covid-19-patients-admitted-to-the-intensive-care-unit-during-the-first-year-of-the-pandemic-in-brazil-a-single-center-retrospective-cohort-study/>).

In addition, meta-analysis conducted on 58 independent studies on COVID-19 showed the similar findings for the biochemical parameters from the current study (<https://www.ncbi.nlm.nih.gov/pmc/articles/PMC8444810/>) indicating that the results from multiple centers were in concordance with the present study (Accordingly, few statements were added in the discussion in Page 16 lines 386 to 388)

In fact, in the present study, the outcome observed in the earlier small cohort with only 17 ICU admitted cases were similar to the current larger cohort. Moreover, the accuracy for predicting the ICU admission increased to 94.2% with inclusion of additional 50 cases in ICU admitted group for cumulative radiological and biochemical parameters than the earlier cohort. The conclusions largely remained the same in both the cases (earlier and now) indicating CORADS along with ferritin and blood urea nitrogen values determines the ICU admission more accurately than considered independent (These changes can be seen in Pages 12 and 13 lines 286 to 303).
